# Regional Intra-Arterial vs. Systemic Chemotherapy for Advanced Pancreatic Cancer: A Systematic Review and Meta-Analysis of Randomized Controlled Trials

**DOI:** 10.1371/journal.pone.0040847

**Published:** 2012-07-18

**Authors:** Fenghua Liu, Yong Tang, Junwei Sun, Zhanna Yuan, Shasha Li, Jun Sheng, He Ren, Jihui Hao

**Affiliations:** Department of Pancreatic Cancer, Key Laboratory of Cancer Prevention and Therapy, Tianjin Medical University Cancer Institute and Hospital, Tianjin, China; University of British Columbia, Canada

## Abstract

**Objective:**

To investigate the efficacy and safety of regional intra-arterial chemotherapy (RIAC) versus systemic chemotherapy for stage III/IV pancreatic cancer.

**Methods:**

Randomized controlled trials of patients with advanced pancreatic cancer treated by regional intra-arterial or systemic chemotherapy were identified using PubMed, ISI, EMBASE, Cochrane Library, Google, Chinese Scientific Journals Database (VIP), and China National Knowledge Infrastructure (CNKI) electronic databases, for all publications dated between 1960 and December 31, 2010. Data was independently extracted by two reviewers. Odds ratios and relative risks were pooled using either fixed- or random-effects models, depending on I^2^ statistic and Q test assessments of heterogeneity. Statistical analysis was performed using RevMan 5.0.

**Results:**

Six randomized controlled trials comprised of 298 patients met the standards for inclusion in the meta-analysis, among 492 articles that were identified. Eight patients achieved complete remission (CR) with regional intra-arterial chemotherapy (RIAC), whereas no patients achieved CR with systemic chemotherapy. Compared with systemic chemotherapy, patients receiving RIAC had superior partial remissions (RR = 1.99, 95% CI: 1.50, 2.65; 58.06% with RIAC and 29.37% with systemic treatment), clinical benefits (RR = 2.34, 95% CI: 1.84, 2.97; 78.06% with RAIC and 29.37% with systemic treatment), total complication rates (RR = 0.72, 95% CI: 0.60, 0.87; 49.03% with RIAC and 71.33% with systemic treatment), and hematological side effects (RR = 0.76, 95% CI: 0.63, 0.91; 60.87% with RIAC and 85.71% with systemic treatment). The median survival time with RIAC (5–21 months) was longer than for systemic chemotherapy (2.7–14 months). Similarly, one year survival rates with RIAC (28.6%−41.2%) were higher than with systemic chemotherapy (0%−12.9%.).

**Conclusion:**

Regional intra-arterial chemotherapy is more effective and has fewer complications than systemic chemotherapy for treating advanced pancreatic cancer.

## Introduction

Pancreatic carcinoma is one of the deadliest cancers. It is the only cancer with relative five-year survival rates that are less than 10%. This is due, in part, to the fact that 80% of patients have advanced unresectable disease at the time of diagnosis [1]. For patients that are not surgical candidates, chemotherapy is typically offered. However, the response rate to standard systemic chemotherapy is very low. Gemcitabine (GEM), the most commonly used first-line drug in pancreatic cancer, only has a 5–15% response rate. In addition, GEM in combination with other anti-cancer drugs does not significantly improve survival [2]. The median survival time for patients with advanced pancreatic cancer is less than 6 months and the 1-year survival rate is less than 18% [3], [4], [5], [6].

Regional chemotherapy, such as regional intra-arterial chemotherapy (RIAC), was introduced as a means of increasing cancer survival rates. Since the 1950s, regional chemotherapy has been evaluated and proven to be effective for some metastatic and localized cancers. Intra-arterial chemotherapy generates high drug concentrations in target areas while maintaining low systemic drug levels. Patients with unresectable colorectal cancer and liver metastasis had high response rates to hepatic arterial infusion (HAI) of chemotherapeutic agents and some tumors also converted to resectability [7], [8]. Clinical trials showed that regional intra-arterial infusion with GEM improved the response and resectability rates for advanced pancreatic cancer (APC), and was well tolerated by patients [9], [10]. However, other research did not show improved tumor response or median survival with regional chemotherapy for pancreatic cancer, thereby casting doubt upon its value as a treatment option for patients with unresectable or recurrent pancreatic cancer [11].

The value of RIAC for treating advanced pancreatic cancer is still unclear. The aim of this study is to clarify the value of RIAC in treating APC by comparing its safety and efficacy with systemic chemotherapy.

**Figure 1:**
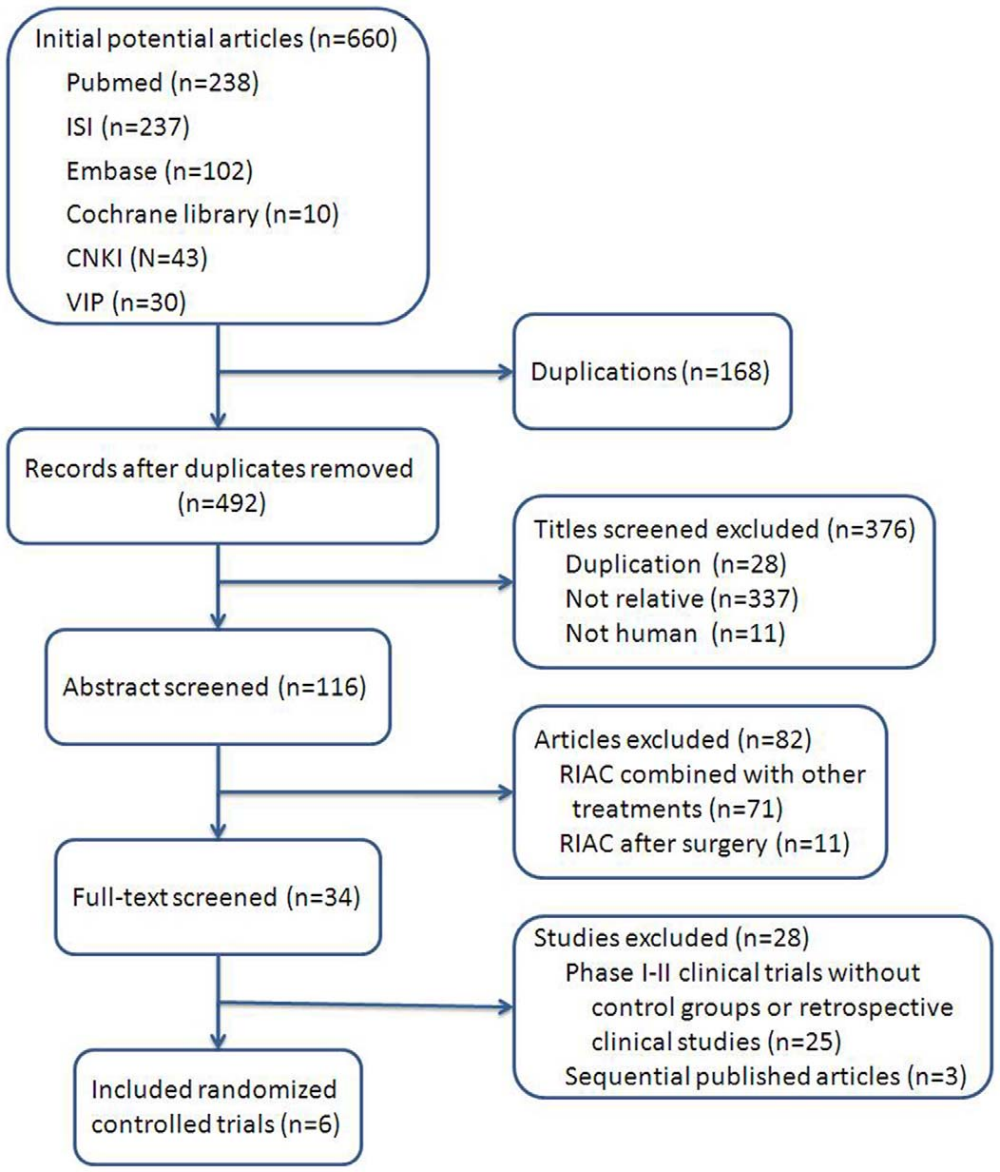
The process of study selecting of articles. The process of searching for articles for inclusion in this systematic review: 660 articles were found and 492 articles remained after removal of duplicates. After review of the complete texts, 6 articles met the inclusion criteria and were included in the meta-analysis.

**Table 1:** Study characteristics.

Study, Country	Samplesize	Diagnosis	Liver metastasis n/N			Mean age (y)	Drugs		GenderNo. M/F	Drug delivery routes		Median or mean survival time (ST)	
	LIAC/C		LIAC		C		LIAC	C		LIAC	C	LIAC	C
Han. 2006, China [12]	70/70	Biopsy proven	NG			60.2	FAM	FAM	110/30	celiac artery splenicartery	intravenous	13.5 m	6.2 m
Shamseddine.2005,America [15]	7/4	Biopsy proven	4/11			NR	GEM	GEM	NR	tumor-feeding arteries	intravenous	5 m	5.6 m
Ji. 2003, China [14]	18/11	pathological/CT/CA199/MRI	8/18	4/11		62.4	MF	MF	16/12	splenic artery, gestroduodenalartery, commonhepatic artery	intravenous	12.5 m (mean ST)	4.8 m (mean ST)
Aigner.1998,Gemany [13]	9/5	pathological/CT/MRI	7/9	4/5		56	MmMC	MmMC	11/3	celiac artery	central venous	8.9 m	2.7 m
Liu. 2008, China [17]	26/27	pathological/CT/MRI	7/26	9/27		61	GP	GP	30/23	superior mesentericartery	intravenous	21 m	14 m
Hong. 2007,China [16]	25/26	pathological/CT/MRI	30/51			NR	GF	GF	NR	tumor-feeding arteries	intravenous	10 m	7.3 m

FAM: adriamycin 40 mg/m^2^, mitomycin (MMC) 6 mg/m^2^, d1; 5-fluorouracil (5-FU), 375 mg/m^2^, d2-6.

GEM: Gemcitabine, 1000 mg/m^2^, day 1, 8.

MF: MMC, 2 mg, d2, 4,6; 5-FU, 750 mg, day 1, 3, 5.

MmMC: Mitomycin C at a total dose of 18 mg/m^2^, day 1–5; mitoxanthrone, 6 mg/m^2^, day 6; cisplatin, 30 mg/m^2^, day 7–8.

GP: Gemcitabine 1000 mg/m^2^, day 1; cisplatin, 50 mg/m^2^, day 1.

GF (GEM+5-FU): GEM 1000 mg/m^2^, day 1; 5-Fu, 600 mg/m^2^, day 1–5.

NR: not reported.

## Materials and Methods

### Search Strategy

Studies were identified by searching PubMed(1966–2010.12), ISI(1997–2010.12), EMBASE(1984–2010.12), Cochrane Library(Issue 1, 2011), Chinese Scientific Journals Database (VIP: 1977–2010.12), and China National Knowledge Infrastructure (CNKI: 1994–2010.12) electronic databases. The keywords“pancreas/pancreatic cancer”, and the Medical Subject Headings (MeSH) “pancreatic neoplasm” and “intra-arterial chemotherapy”, were combined with exploded index terms and synonyms for searches of keywords, abstracts, and titles. The search strategy used was: 1# pancreatic cancer, 2# pancreas cancer; 3# pancreatic carcinoma; 4# pancreas carcinoma; 5# MeSH descriptor “pancreatic neoplasm” explode all trees; 6# regional arterial infusion; 7# arterial perfusion; 8# regional chemotherapy; 9# intra-arterial chemotherapy; 10# trans-artery chemotherapy; 11# hepatic arterial infusion; 12# celiac artery; 13# drug delivery pathway; 14# splenic artery; 15# regional treatment;16# locally intra-arterial infusion; 17#: 1# or 2# or 3# or 4# or 5#; 18#: 6# or 7# or 8# or 9# or 10# or 11# or 12# or 13# or 14# or 15# or 16#; 19#: 17# and 18#.

**Table 2:** Evaluation of the quality of RCTs included in the meta-analysis.

Risk of bias	Random sequence generation	Allocation concealment	Blinding	Incomplete outcome data	Selective reporting	Other bias
Aigner.1998	Unclear	Unclear	High risk	Low risk	Low risk	Low risk
Han. 2006	Unclear	Unclear	High risk	Low risk	Low risk	Low risk
Ji.2003	Unclear	Unclear	High risk	Low risk	Low risk	Low risk
Hong. 2007	High risk	Unclear	High risk	Low risk	Low risk	Low risk
Liu. 2008	Low risk	Unclear	High risk	Low risk	Low risk	Low risk
Shamseddine. 2005	High risk	Unclear	High risk	Low risk	Low risk	Low risk

**Figure 2:**
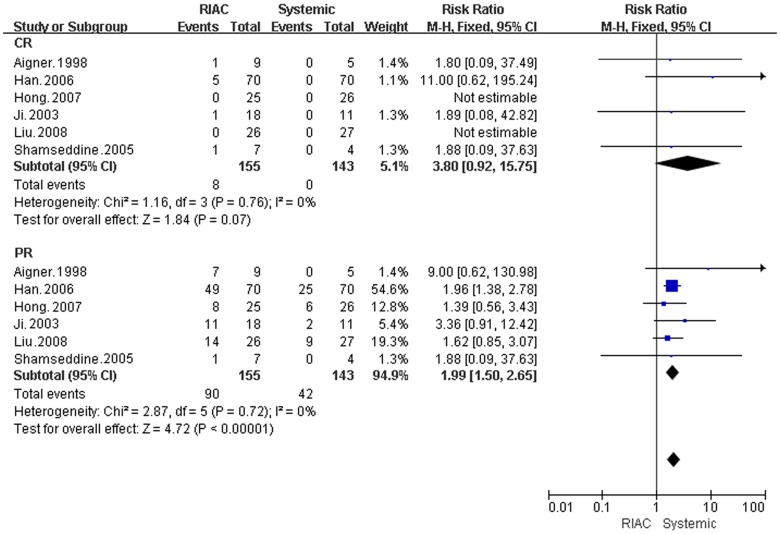
Meta-analysis of CR and PR. Diamonds represent pooled effects. CR = complete remission, PR = partial remission.

**Figure 3:**
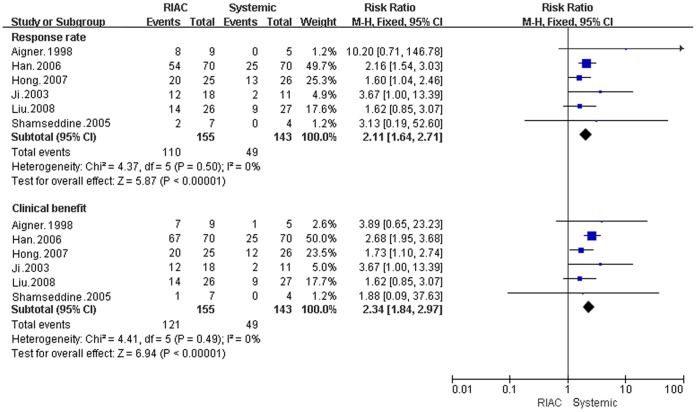
Meta-analysis of response rates and clinical benefits. Diamonds represent pooled effects.

### Selection Criterion

Only prospective randomized controlled trials (RCT) were selected for inclusion in the study. Patients with APC, regardless of the existence of liver or peritoneal metastasis, were included in this study. Patients were treated with either RIAC (via the cancer feeding artery, hepatic artery, celiac artery, gestroduodenal artery, superior mesenteric artery, common hepatic artery, splenic artery, or other regional arteries, with or without regional embolization), or systemic intravenous chemotherapy (via central or peripheral veins). Systematic reviews of randomized controlled trials were included. Comparative studies (historical and non-randomized) and those where pancreatic cancer was not confirmed by pathology or by imaging (such as CT, MRI) were excluded.

**Figure 4:**
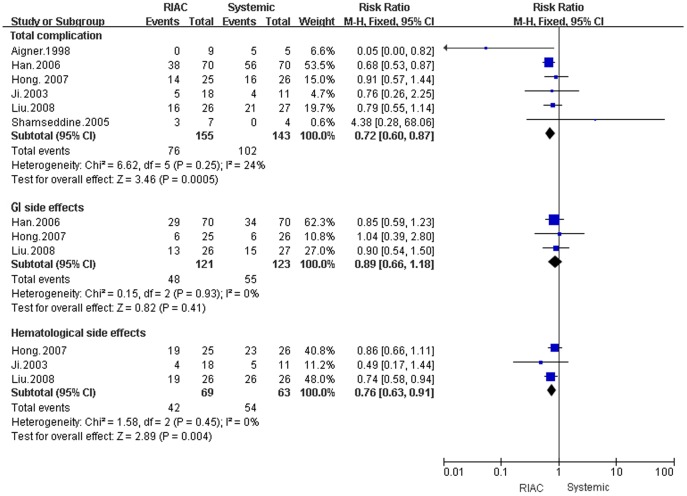
Meta-analysis of the incidence of complications using Regional Intra-Arterial Chemotherapy or systemic administration of chemotherapeutics. Diamonds represent pooled effects. Total complications and hematological system side effects differed between Regional Intra-Arterial Chemotherapy and systemically treated patients (P = 0.01). Gastrointestinal system side effects did not differ between treatment groups (P = 0.35).

### Quality Assessment

We assessed the methodological quality of each RCT using the criteria outlined in the Cochrane Handbook for Systematic Reviews of Interventions (CHSRI). The following CHSRI components were assessed by two reviewers (FHL and YT): adequate generation of random sequences (selection bias), allocation concealment (selection bias), blinding (performance bias and detection bias), incomplete outcome data (attrition bias), selective reporting (reporting bias), source of funding bias, baseline imbalance, and early stoppage. These components were graded as low risk, high risk or unclear. Methodological information for the assessment of validity was extracted by two reviewers (FHL and JWS).

### Data Extraction and Analysis

A pre-designed data extraction table was used to extract the characteristic data of the eligible articles by two independent reviewers (FHL and JWS), with reviewer discrepancies resolved by a supervisor (YT). The following information was extracted from each report: authors, time of publication, patient eligibility criteria, number of patients in the RIAC and systemic treatment groups, gender, and route of drug administration. Major end-points extracted for each report were: complete remission (CR), partial remission (PR), clinical benefit, pain-control, and complication rate. The end-point definitions used were: CR was complete disappearance of liver metastasis or the primary pancreatic cancer; PR was the diameter of all liver metastases with no evidence of new or progressive lesions; no change (NC) was a <50% reduction or a <25% increase in diameter; progressive disease (PD) was a >25% increase in diameter of one or more liver metastases, or the occurrence of new liver metastases. Treatments were considered effective for patients achieving CR or PR. Patients achieving pain relief or an increase of (KPS) or (PS) were classified as receiving clinical benefits [12]. Side effects of interest mainly involved the hematological (leucopenia, thrombocytopenia or anemia) and gastrointestinal systems (nausea, vomiting or duodenal ulcer). Embolization, thrombophlebitis and catheter displacement were additional complications of interest.

Statistical heterogeneity among studies was assessed using Q and I^2^ statistics. Meta-analysis of studies with an acceptable level of heterogeneity (p>0.1, or p≤0.1 but I^2^≤50%) was conducted using a fixed-effects model. A random-effects model was used for studies where significant heterogeneity was found (p≤0.1, I^2^>50%). Parameters that were analyzed included the number of the patients, major end-points (CR, PR, clinical benefit, pain relief, response rate, and complication rate), and digestive and hematological side-effects. Statistical significance was P≤0.05. Data from RCTs meeting inclusion criteria were analyzed with Revman 5.0 (RevMan 5.0.23; Cochrane Collaboration, Oxford, UK).

## Results

### Study Characteristics

The database search strategy initially retrieved 660 publications, and 168 were eliminated due to duplication (Table 1). English [12], [13], [14], [15] (n = 4) and Chinese [16], [17] (n = 2) language publications met the study’s inclusion criteria. These publications included patients receiving RIAC (n = 155) and systemic chemotherapy (n = 143) (Figure 1). No systematic review or meta-analysis was found.

### Quality Assessment

One study explicitly stated that patients were selected using a random envelope process [17], whereas the other studies did not indicate that random selection was employed. One study could not be assessed for adequate sequence generation [15]. No studies were blinded and there was no evidence of allocation concealment. No funding biases were evident for any studies. There were no studies with incomplete outcome data, early stoppage bias, or baseline imbalances. The risks of bias and corresponding ratios were summarized (Table 2).

### Efficacy

#### Overall survival

Five studies [12], [13], [15], [16], [17] reported that RIAC median survival times (5–21 months) were longer than for systemic chemotherapy (2.7–14 months).One of these RCTs [13], [16] had survival times in the systemic group of 3.5, 6, 11, 13, 20 months, whereas they were 15, 16, 25, 31, 33, 39, 47, 56, 59 months, for RIAC patients. One of these RCTs [16]had longer RIAC survivorship than for systemic chemotherapy for 6-month (84.0% vs. 52.5%), 9-month (76.0% vs. 38.9%), 1-year overall survival (OS) (48% vs.14.6%) and median survival times (10.0 m vs.7.3 m). These two RCTs demonstrated that patients receiving RIAC had greater 1-year OS (41.2%−28.6%) compared with systemic chemotherapy (0–12.9%). We had tried to contact with authors, but we were unable to obtain further information for inclusion in our meta-analysis.

#### Complete remission and partial remission

Six studies (298 patients) were selected for the meta-analysis. The RIAC and systemic groups did not differ significantly for CR (RR = 3.80, 95% CI: 0.92, 15.75). However, unlike the patients treated with systemic therapy, there were patients that achieved CR with RIAC (n = 8) [12], [13], [14], [17], and one patient became eligible for R0-resection [13]. In addition, patients treated with RIAC (n = 90; 58.06%), had better PR than did patients treated with systemic chemotherapy (n = 42; 29.37%) (RR = 1.99, 95% CI: 1.50, 2.65) (Figure 2).

#### Clinical benefit and response rates

Patients in the RIAC group received more clinical benefits (78.06% )(RR = 2.34, 95% CI: 1.84, 2.97) and better response rates (70.97%) (RR = 2.11, 95% CI: 1.64, 2.71) than the systemic group (29.37% and 34.27%, respectively) (Figure 3).

### Complications

#### Gastrointestinal and hematological side effects

The overall incidence of complications was lower in RIAC patients (49.03%) than those receiving systemic chemotherapy (71.33%) (RR = 0.72, 95% CI: 0.60, 0.87). No deaths due to drug toxicity were reported, and there were no differences in gastrointestinal side effects (RR = 0.82, 95% CI: 0.66, 1.18). However, the RIAC group (60.87%) had fewer hematological side effects than the systemic group (85.71%) (RR = 0.76, 95% CI: 0.63, 0.91) (Figure 4).

#### Catheter complications

Catheter displacement was found in one patient [16]. No patients developed embolization or thrombophlebitis associated with catheter implantation.

## Discussion

Our meta-analysis of six prospective RCTs included 298 patients with APC. Compared with systemic chemotherapy for treatment of APC, RIAC resulted in higher PR, clinical benefits, and response rates with fewer complications.

### Efficacy

Pancreatic cancer is relatively resistant to chemotherapy. GEM is the first-line chemotherapy drug for APC (5–15% efficiency). Combinations of GEM with other anti-cancer drugs do not significantly improve the survival of patients (OS was 5.6–8.2 months) [18], [19], [20], [21].

The effect of chemotherapy is concentration dependent. Intra-arterial infusion generates higher drug concentrations within targeted regions with lower systemic drug concentrations [22], [23], [24]. This reduces the risks of systemic toxicity while increasing target tissue drug efficacy. Shamseddine [15] measured plasma concentrations of dFdU (2′-2′-Difluorodeoxyuridine; the deammoniated metabolic product of Gemcitabine) 30 minutes and 270 minutes after administration. The concentration of dFdU in patients receiving systemic treatment was higher than for RIAC patients (RR 30 min = −5.10, 95% CI: −8.02, −2.17; RR 270 min = −173.36, 95% CI: −263.80, −82.93; P<0.05). Yang [25] found reduced hepatic metastasis (RR = 0.46, 95% CI: 0.31–0.69) and longer mean survival times (RR = 0.44, 95% CI: 0.28–0.68). in RIAC treated mice with APC. High response rates (complete response 8.7% and partial response 65.2%) in a descriptive study of 31 patients with advanced pancreatic cancer that received RIAC were accompanied by 1-, 2- and 3-year survival rates of 90.9, 42.8 and 18.3% [26], respectively. Similarly, Nakchbandi [27] demonstrated improved median survival times (12.7 months) for patients with metastatic pancreatic cancer treated with RIAC. This study identified two RCTs [13], [16] where RIAC improved the 1-year OS (41.2%−28.6%), compared with systemic chemotherapy (0−12.9%).

### Complications

Side effects of chemotherapy were dependent upon the drug regimen used and the drug administration route. Common side effects included nausea, vomiting, hair loss, and bone marrow suppression. This meta-analysis result showed that overall complications for RIAC (49.03%) were less than for systemic chemotherapy (71.33%), and there were no treatment associated deaths [14], [16], [17]. However, the only statistically significant difference was for hematologic side effects (RR = 0.76, 95% CI: 0.63, 0.91; 60.87% for RIAC and 85.71% for systemically treated individuals) [12], [16], [17], and only 0.1% patients experienced severe complications. Pharmacokinetic data indicated that systemic concentrations of the drug were lower for RIAC than with systemic chemotherapy, which could be the basis for the reduction in complications for RIAC [22], [28], [29], [30], [31].

Although the value of regional chemotherapy has been demonstrated, expansion of its clinical use is constrained by some drawbacks. For example, RIAC is generally much more difficult to administer than systemic chemotherapy; it is an invasive procedure with increased time and costs of hospitalization; as well as having increased local complications. However, RIAC has superior clinical benefits and fewer systemic complications. This makes RIAC a good strategy for treatment of APC, as well as a good option for palliative or neoadjuvant therapy, especially in patients who don’t respond to standardized therapy.

### Limitations

This meta-analysis has limitations. Bias may have been introduced because nonpublished data was not included and the relatively few numbers of patients in our study. Larger and more methodologically rigorous clinical trials are needed to confirm these findings.
